# Sponge Mass Mortalities in a Warming Mediterranean Sea: Are Cyanobacteria-Harboring Species Worse Off?

**DOI:** 10.1371/journal.pone.0020211

**Published:** 2011-06-01

**Authors:** Emma Cebrian, Maria Jesus Uriz, Joaquim Garrabou, Enric Ballesteros

**Affiliations:** 1 Universitat de Girona, Facultat de Ciències, Departament de Ciències Ambientals, Girona, Spain; 2 Centre d'Estudis Avançats de Blanes-CSIC, Blanes, Girona, Spain; 3 Institut de Ciències del Mar-CSIC, Barcelona, Spain; 4 Centre d'Estudis Avançats de Blanes-CSIC, Blanes, Girona, Spain; National Institute of Water & Atmospheric Research, New Zealand

## Abstract

Mass mortality events are increasing dramatically in all coastal marine environments. Determining the underlying causes of mass mortality events has proven difficult in the past because of the lack of prior quantitative data on populations and environmental variables. Four-year surveys of two shallow-water sponge species, *Ircinia fasciculata* and *Sarcotragus spinosulum,* were carried out in the western Mediterranean Sea. These surveys provided evidence of two severe sponge die-offs (total mortality ranging from 80 to 95% of specimens) occurring in the summers of 2008 and 2009. These events primarily affected *I. fasciculata*, which hosts both phototrophic and heterotrophic microsymbionts, while they did not affect *S. spinosulum*, which harbors only heterotrophic bacteria. We observed a significant positive correlation between the percentage of injured *I. fasciculata* specimens and exposure time to elevated temperature conditions in all populations, suggesting a key role of temperature in triggering mortality events. A comparative ultrastructural study of injured and healthy *I. fasciculata* specimens showed that cyanobacteria disappeared from injured specimens, which suggests that cyanobacterial decay could be involved in *I. fasciculata* mortality. A laboratory experiment confirmed that the cyanobacteria harbored by *I. fasciculata* displayed a significant reduction in photosynthetic efficiency in the highest temperature treatment. The sponge disease reported here led to a severe decrease in the abundance of the surveyed populations. It represents one of the most dramatic mass mortality events to date in the Mediterranean Sea.

## Introduction

Global warming is an emerging threat to ecosystems worldwide [1]. Marine coastal environments, especially in the tropics, are suffering dramatic increases in mass mortality events and diseases associated to ocean warming [2], [3]. In the Caribbean alone, 28 major epidemics have been documented in a wide variety of taxa since 1980 [2]. Information on temperate seas is far less abundant, although reports point to a rising trend in major epidemics [2], [3].

In the temperate western Mediterranean Sea, two major mass mortality events of sessile epibenthic invertebrates occurred in 1999 and 2003 [4]. They involved a wide range of species (approximately 30 species from 5 phyla) and covered an extensive geographical area (1000 km of coastline) [4]–[6]. In addition to these two major events, other mass mortality events that affected fewer species or were geographically restricted have been recorded in the Mediterranean Sea during the last decades [7]–[11]. In general, these events were concomitant with anomalous high temperature conditions [4]. The occurrence of these mortality events has raised concern among scientists and managers about the potential effects of global warming on Mediterranean biodiversity [4], [12].

To date, the effects of mass mortality events have only been quantified at the population level in gorgonian species (e.g., [4]–[5], [13]–[15]). The lack of baseline data hinders quantification of the extent of mortality on other dramatically affected organisms, such as sponges. In addition, few studies have examined the biological mechanisms causing mass mortality events in the Mediterranean (but see [11], [16]–[17]). The study of sponge diseases is particularly challenging because sponges host a high density and diversity of microorganisms, including heterotrophic bacteria, cyanobacteria, unicellular algae, diatoms, viruses, fungi and archaea [18]; reviewed in [19].

In late summers of 2008 and 2009, extensive die-offs of shallow water sponge populations were observed in Cabrera National Park and Scandola Marine Reserve. The species with the highest proportion of die-offs was *Ircinia fasciculata,* which hosts cyanobacteria and heterotrophic bacteria. *Sarcotragus spinosulum*, another keratose sponge that harbors only heterotrophic bacteria, was hardly affected. Because the mortality outbreak was concomitant with high temperature conditions, the working hypothesis was that high temperatures may induce a breakdown of the cyanobacteria-sponge symbiosis, causing *I. fasciculata* mortality.

The aims of the present study were (i) for the first time, to provide accurate quantitative data (before and after the events) on Mediterranean sponge die-offs and comparatively evaluate their impact on two keratose sponges with and without symbiotic cyanobacteria (*I. fasciculata* and *S. spinosulum,* respectively) in Cabrera and Scandola; (ii) to assess the relationship between seawater temperature, sponge habitat and sponge mortality; and (iii) to experimentally determine the effect of elevated temperatures on the photosynthetic efficiency of symbiotic cyanobacteria hosted by *I. fasciculata*.

## Materials and Methods

### Study sites

**Figure 1:**
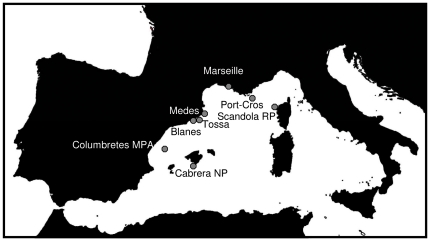
The western Mediterranean Sea, showing the surveyed localities of *Ircinia fasciculata*.

Quantitative surveys on the sponge mortality were carried out in two Marine Protected Areas in the western Mediterranean Sea: Cabrera National Park (Balearic Islands, Spain, hereafter Cabrera NP) and Reserve Naturelle de Scandola (Corsica, France, hereafter Scandola RN) (Fig. 1). Quantitative assessments of sponge population's status started in 2007 in Cabrera NP and were extended in 2008 to Scandola RN till late summer 2010. After the first observation of mortality signs in October 2008, qualitative surveys (presence/absence) of mortality (presence of partial or total necrosis) were carried out in six other zones encompassing the Spanish and French coasts (Table 1; Fig. 1). Finally, sponge mortality recorded by other research teams in several areas of western Mediterranean was also compiled to assess the geographical extent of the sponge die-offs (Table 1).

**Table 1:** Localities and geographical position where the presence (+) or absence (−) of mass mortality was assessed.

Locality	coordinates	*I. fasciculate* die-off
*Blanes (2 sites)	41.674N ; 2.74E	−
*Tossa de Mar (1 site)	41.720N ; 2.93E	−
*Medes Islands MPA (3 sites)	42.047N ; 3.22E	−
*Cabrera NP (3 sites)	39.140N ; 2.94E	+
*Scandola RP (1 site)	42.356N ; 8.56E	+
*Marseille (4 sites)	43.297N ; 5.38E	−
*Port-Cros (3 sites)	43.009N ; 6.38E	−
*Columbretes MPA (6 sites)	39.055N ; 0.44E	−
**Granada Coast	36.728N ; 3.44W	+
**Chafarinas Islands	35.005N ; 2.25W	+

* Present study

** Maldonado et al. 2010.

### Target species

The species selected for the survey were *Ircinia fasciculata* (Esper 1794) and *Sarcotragus spinosulum* (Schmidt 1862). Both species are abundant in shallow waters of the western Mediterranean [20], are phylogenetically close (*Sarcotragus* was considered a subgenus of *Ircinia* until 2002 [21]), and belong to the category of “bacteriosponges” because they harbor a high proportion of symbiotic microbes relative to sponge biomass [22]. *I. fasciculata* harbors both cyanobacteria and heterotrophic bacteria, while *S. spinosulum* hosts only heterotrophic bacteria. Both species inhabit the photophilic assemblages and are usually restricted to the first 20 m of depth.

### Sponge survey and mortality assessment

Several populations of *I. fasciculata* and *S. spinosulum* were monitored for sponge densities and injury incidence in July and October (before and after the period of warmest temperatures) by placing 20 quadrats of 2500 cm^2^ haphazardly between 10 and 15 m depth at each surveyed site. We counted the total number of specimens and those suffering partial or total mortality (when specimens were totally dead, the skeletons remained *in situ* for ca. 1 month after the sponge detached from the substratum [23], [24]). The surveys were carried out in three sites in Cabrera NP from July 2007 to October 2010 and in one site in Scandola RN from October 2008 to October 2010. In the remaining surveyed areas we annually surveyed only for the presence or absence of mortality after summer (Table 1).

**Figure 2:**
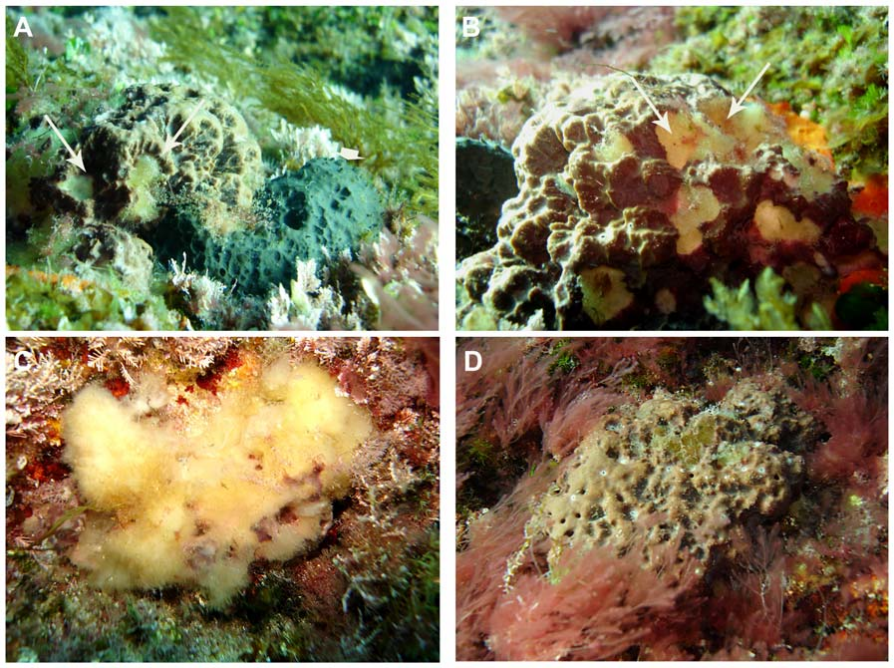
Aspects of *Ircinia fasciculata* mortality. a) First stage of the affectation on *I. fasciculata* (arrow head) compared with healthy *S. spinosulum* (arrow), b) small whitish spots on the *I. fasciculata* surface, c) completely dead *I. fasciculata* with the skeleton (spongin network) free of cellular material, (d) white bacterial veil on unhealthy *Sarcotragus spinosulum* surface.

As affected sponges we considered those individuals displaying small whitish spots on the sponge surface characteristics of the early sponge disease; as well as those specimens displaying one or more necrotic zones, where the skeleton (spongin network) was free of cellular material (see Results) characteristic of the later disease stages (see Results Fig. 2).

The density and percentage of injured sponges in Cabrera NP was analyzed for both species by two-way ANOVA with time (fixed) and site (random) as factors. Differences in the density and percentage of injured sponges in Scandola RN across time were analyzed by one-way ANOVA. Tukey tests were used for post-hoc comparisons. Prior to analysis, the homogeneity of variances was verified with Cochran's test. The analyses were carried out by using the Statistica 8.0 software.

### Ultrastructural study

Healthy and injured tissues from the same specimen of *I. fasciculata* were examined by transmission electron microscopy (TEM) to assess the main ultracellular changes in sponge cells, cyanobacteria, and heterotrophic bacteria, associated with the sponge disease. For TEM observation, small samples (2–3 mm in diameter) were fixed in a solution consisting of 1% OsO_4_ and 2% glutaraldehyde in 0.4 M sodium cacodylate buffer (pH 6.4) with 10% sucrose for 2 h at 4°C [25]. The samples were then rinsed in the buffer solution, dehydrated in a graded ethanol series to 70% and embedded in Spurr's resin. Semi-thin sections were stained with uranyl acetate and lead citrate and observed through a Hitachi H-600 transmission electron microscope (Microscopy Unit of the Scientific and Technical Services at the University of Barcelona).

### Experimental approach to assess the effects of temperature on the photochemical efficiency of I. fasciculata cyanobacteria symbionts

Variation on the cyanobacteria photochemical efficiency under several temperatures was measured in a set of laboratory experiments. Eighteen specimens (ca. 25 cm in diameter) of *I. fasciculata* were randomly collected in Tossa de Mar, Girona, Spain, at a depth of 10 m in January 2010, when the water temperature was 14°C. The samples were transported in aerated seawater under low-light conditions at 14°C to the CEAB aquarium facilities, where the experiments were conducted. The specimens of *I. fasciculata* were randomly transferred to three experimental tanks (50 L): one control and two temperature treatments. The experiment temperature treatments were: “control” (16°C), “normal summer T” (23°C) and “extreme summer T” (27°C) according to the T values recorded in the NW Mediterranean [26]. Light was provided by several metal halide lamps (Philips, HPIT 40 W) at an intensity of 400 µmol photon m^−2^ s^−1^ to simulate the irradiance conditions of the sponge habitat [27]. Irradiance was measured using a spherical Li-Cor underwater quantum sensor (LI-193SA). Sponges were maintained for a 48 h adaptation period under low (16°C) temperature conditions in the three tanks. After the adaptation period, we progressively increased the temperature (1°C every 12 h) in the treatment tanks for 8 days until the “medium” (23°C) and for 10 days until the “extreme” (27°C) temperatures were reached, using aquarium heaters connected to electronic controllers (±0.1°C). Temperatures were subsequently maintained at 16°C, 23°C and 27°C for 48 h. The effect of temperature on cyanobacteria was estimated by measuring the symbionts' photochemical efficiency by means of chlorophyll fluorescence parameters (effective quantum yield: Φ_PSII_ quantum yield and photosynthetic electron transfer: ETR  =  Φ_PSII_ *PAR*0.5*AF, where Φ_PSII_ is the effective quantum yield of Photosystem II and PAR is the photosynthetically available irradiance reaching the sample) using a PAM fluorometer (DIVING-PAM, Walz, Germany) [28].

One-way ANOVA on ETR and Φ_PSII_ values obtained during the first day of the experiment was carried out to verify that sponges undergoing different treatments started at similar photosynthetic activity conditions. To assess the effects of increasing temperatures on cyanobacteria photosynthetic activity, one-way ANOVA was used to test for differences in ETR and Φ_PSII_ values obtained for each treatment at the end of the experiment. Prior to analysis, the homogeneity of variances was verified by Cochran's test. These analyses were carried out with the Statistica 8.0 program.

### Temperature conditions

High-resolution (hourly records) temperature series were obtained at Cabrera NP and Scandola RN at 15 m depth since 2007 and 2008 respectively. Temperature was recorded *in situ* by Stowaway Tidbits, autonomous sensors (0.2 °C precision, 0.15 °C resolution), and recovered annually by divers (see [26] for more details). For the data analysis we considered the period from August 1 to September 30 as the summer period. The average mean and maximum temperatures for each summer period were calculated. Moreover, given the short time interval between measurements (1 h), the data set allowed the calculation of the percentage of time spent above the following temperature thresholds: 23, 24, 25 and 26°C. We used these different descriptors of summer temperature conditions to explore the relationship between the mortality on sponge populations and temperature.

### Relationship between mortality and temperature conditions

A regression analysis between the percentage of time above the maximum temperature threshold during the summer period and the percentage of injured *I. fasciculata* specimens was performed to explore the potential role of the high temperatures in the *I. fasciculata* die-off. As thermal regimes are particular for each study area, thresholds selected were those showing the highest differences between mortality and non mortality years in Cabrera NP (>26°C) and Scandola RN (25°C) (see results). The analysis was performed with Statistica 8.0.

## Results

### Morphological and structural features of the sponge disease

The disease in *I. fasciculata* specimens was initially characterized by small yellowish spots that soon transformed into small zones of necrotic tissue, and then enlarged and fused to form, rounded areas of necrotic ectosome in a few days (Fig. 2a, b). Soon after, the necrotic, rounded areas interconnected, forming large areas of exposed skeleton deprived of tissue. Some partially affected specimens still surviving at the end of the summer were able to heal these wounds in few weeks, while others progressively amassed necrotic tissue and completely died (Fig. 2c). Skeletons of completely dead specimens were eventually detached by storms, leaving a conspicuous mark of naked substrata in the rock that lasted several months.

In contrast, the few injured *S. spinosulum* never displayed the above described pattern, but showed a typical bacterial veil covering the sponge ectosome (Fig 2d), as frequently reported in rotten surfaces of many other sponges (e.g., [29], [30]).

**Figure 3:**
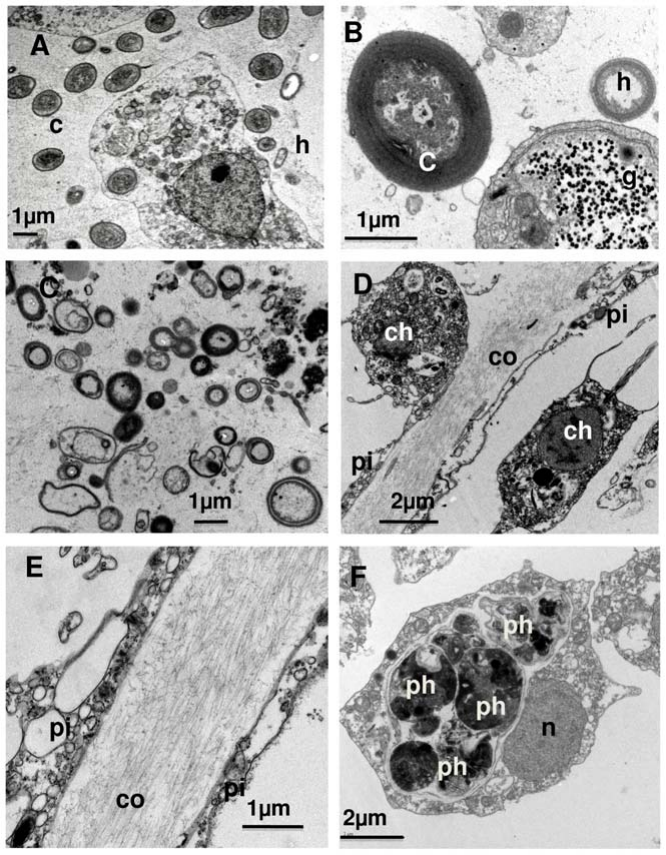
Several ultrastructural aspects of a healthy *Ircinia fasciculata*. A) Archeocyte of a subectosomal zone, surrounded by numerous cyanobacteria (c) and some heterotrophic bacteria (h); B) Detail of a healthy cyanobacteria, close to an archeocyte plenty of glycogen rosettes (g); C) “symbiotic” bacterial community, typically found in the sponge; D) collagen layer (co) between endopinacocytes (pi) separating two choanocyte chambers (ch: choanocytes); E) collagen bundles, surrounded by endopinacocytes (pi); F) basal section of a choanocyte, full of heterogeneous phagosomes (ph), which is characteristic of the species (n: nucleus).

TEM observation of spotted (death) areas, zones in close vicinity to the spots, and healthy zones of *I. fasciculata* showed remarkable differences at an ultrastructural level. Healthy zones contained healthy cyanobacteria (Fig. 3a, b), heterotrophic “symbiotic” bacteria (Fig 3c), abundant collagen bundles (Fig. 3d, e) and choanocytes with huge amounts of phagosomes (Fig. 3f). Copepod exoskeletons, semi-digested heterotrophic bacteria, and muscle tissue, were among the identifiable materials within choanocyte phagosomes.

**Figure 4:**
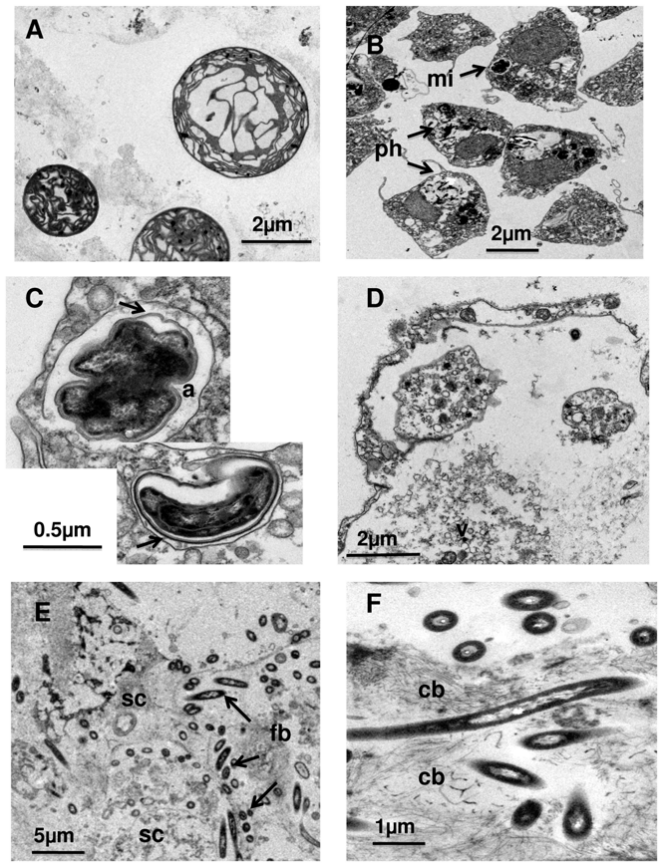
Several ultrastructural aspects of unhealthy specimens of *Ircinia fasciculata*. A, B, C) Zone in the vicinity of a pustule (whitish spot): A) cyanobacteria under several degradation stages; B) section of a degenerating choanocyte chamber with multiple phagosomes (ph) and an unknown microorganism (mi); C) closer view of the “rare” microorganism, which divides within cell vacuoles (a) and shows a membrane complex (arrows) and an irregular, dark inner zone; D, E, F). Zone corresponding to a pustule (died whitish spot): D) completely degenerated cells with multiple released vesicles (v); E) Abundant foreign bacteria (fb), similar to the morphotype reported to consume on the skeleton of dead sponges (Vacelet et al. 1994), among completely degenerated sponge cells (sc); F) Close view of this particular bacterium, which is always associated to the collagen bundles (cb).

The sponge tissue in close vicinity to the necrotic zones contained both choanocytes and archeocytes, plenty of phagocytosed cell debris, and empty vesicles. The cell nuclei were irregular in shape, with a poorly defined nuclear membrane. Cyanobacteria were always degraded, showing a cell volume that was twice to three times the volume of normal cyanobacteria (Fig. 4 a, b). Collagen fibrils were equally abundant in affected and healthy tissue although showed a poorer organization in the former (Fig. 4f). A non-identified microorganism, with a multilayered membrane was quite frequent in cellular vacuoles of disorganized choanocytes, where it divided (Fig. 4c). However, it could not be related unambiguously to the disease.

The necrotic zones (whitish spots) showed total tissue destruction, absence of cyanobacteria, numerous vesicles, broken membranes, and collagen remains (Fig. 4d). A characteristic bacterium invaded the collagen areas (Fig. 4e, f). This bacteria was similar to that described tunneling spongin fibers in free skeletons of dead keratose, species (e.g. [31]), and have also been observed perforating the spongin basal plate of several healthy encrusting species such as *Crambe crambe* (authors obs.).

No specific pathogen involved in the disease could be identified in the ultrastructural study. Cell destruction as indicated by broken membranes and cell disorganization, degeneration and then absence of cyanobacteria and the presence of heterotrophic bacteria similar in ultrastructure to those present in healthy tissue were the main features of the affected sponge tissue.

### Impact of the sponge disease

**Figure 5:**
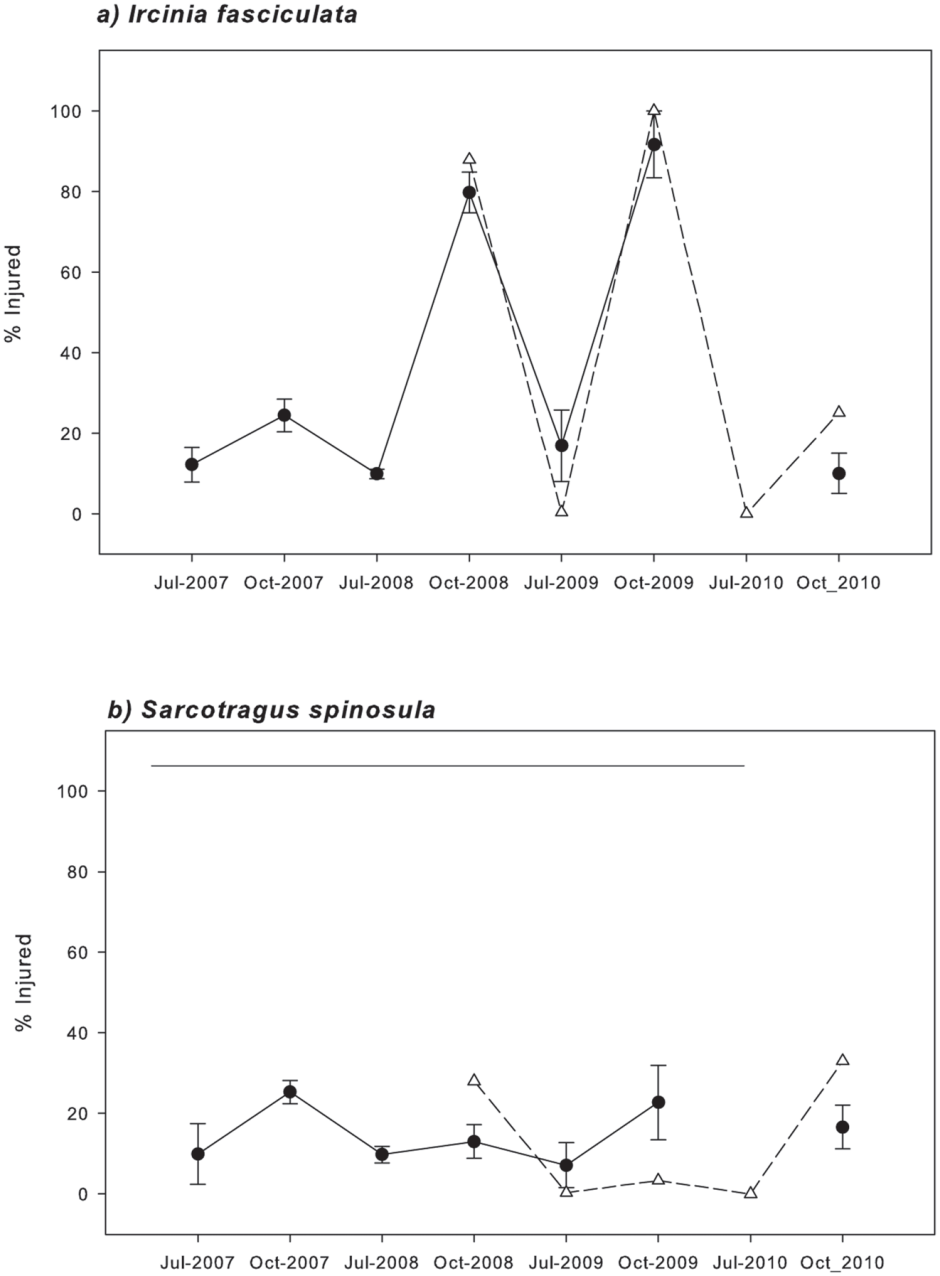
Mean percentages of injured *Ircinia fasciculata* (a) and *Sarcotragus spinosulum* (b) specimens recorded in Cabrera NP (circles) and Scandola RN (triangles) during the monitoring period. Bars represent standard errors. Mean concentrations, which were not significantly different in a Tukey test, are joined by horizontal lines.

The percentage of injured *I. fasciculata* specimens peaked up to 80–100% of the surveyed individuals for both areas, Cabrera NP and Scandola RN, in October 2008 and again in October 2009 (Fig. 5a, Table 2). In contrast, during the rest of the study period, injured individuals never exceeded 30% (Fig. 5a).

**Table 2:** Two-way ANOVA results for Cabrera NP *Ircinia fasciculata* and *Sarcotragus spinosulum* densities and % of injured specimens, with site (random) and time (fixed) factors.

Cabrera NP			
***I. fasciculata*** ** density**			
	*MS*	*F*	*Post-hoc (Tukey-test)*
Site	0.307	3.01	
Time	3.071	30.16**	J07 = O07 = J08 = O08>J09 = O09 = O10
Site*Time	0.67	0.65	
***S. spinosulum*** ** density**			
	*MS*	*F*	*Post-hoc (Tukey-test)*
Site	0.218	2.851	
Time	0.522	6.823*	(O07 = J08)>O09
Site*Time	0.577	0.744	
**% ** ***I. fasciculata*** ** Injured**			
	*MS*	*F*	*Post-hoc (Tukey-test)*
Site	17.00	0.09	
Time	7672	42.05**	J07 = O07 = J08 = J09<O08 = O09 = O10
Site*Time	237	1.30	
**% ** ***S. spinosulum*** ** Injured**			
	*MS*	*F*	*Post-hoc (Tukey-test)*
Site	295.9	2.868	
Time	315.6	3.059	
Site*Time	137.7	1.334	

Scandola RP			
**Density**			
	**MS**	**F**	**Post-hoc (Tukey-test)**
*I. fasciculata*	4.069	127.2.5**	O08>J09 = O09 = J10 = O10
*S. spinosulum*	0.222	0.416	
**% Injured**			
*I. fasciculata*	7140	8033**	O08 = O09
*S. spinosulum*	785.9	4.296	

ANOVA results for Scandola RP *Ircinia fasciculata* and *Sarcotragus spinosulum* densities and % of injured specimens across time.

For *S. spinosulum*, the percentage of injured specimens varied between 10% and 30% in both areas from 2007 to 2010, but differences over time were not significant in any area (Fig. 5b, Table 2).

**Figure 6:**
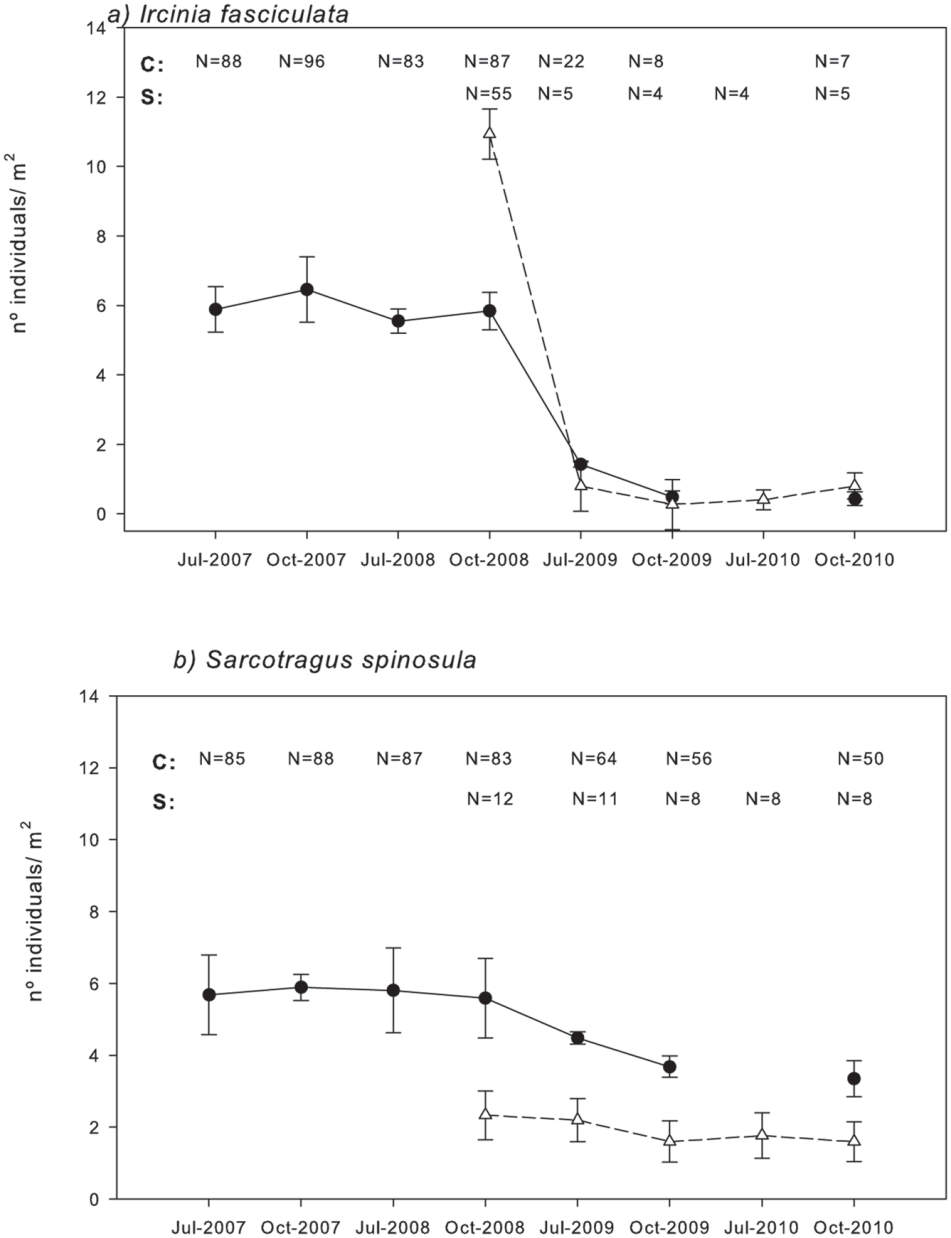
Mean densities of *Ircinia fasciculata* (a) and *Sarcotragus spinosulum* (b) recorded in Cabrera NP (circles, C) and Scandola RN (triangles, S) during the monitoring period. Bars represent standard errors. Mean concentrations, which were not significantly different in a Tukey test, are joined by horizontal lines. N =  number of specimens sampled.

In both sites, densities of *I. fasciculata* decreased dramatically from around 10 (in Scandola RN) and 7 (in Cabrera NP) specimens·m^−2^ before the mortality event (summer 2008) to less than 1 specimens·m^−2^ at the end of autumn 2010 (Fig. 6a). The ANOVA analyses showed that time had a significant effect on *I. fasciculata* densities in Cabrera NP and Scandola RN, which were significantly lower in July and October 2009 and July 2010, after the first outbreak (Fig. 6a, Table 2). In Cabrera NP, where three sites were monitored, no significant differences in *I. fasciculata* densities were observed between sites (populations); neither was the interaction between locality and time significant (Table 2), indicating that the pattern of density losses was similar everywhere (Table 2).

*S. spinosulum* densities in Cabrera NP ranged from 6.8 to 3.35 specimens·m^−2^ from July 2008 to October 2010. Time had a significant effect on *S. spinosulum* densities; the densities in October 2009 and July 2010 were lower than those recorded in October 2007 and July 2008 (Fig. 6b, Table 2). In Cabrera NP, no significant differences in *S. spinosulum* densities were observed between sites, and there was no interaction between locality and time, which indicated a similar density pattern in the three localities (Table 2). Conversely, in Scandola RN, time had no significant effect, even though densities decreased from 2.33 to 1.60 specimens·m^−2^ between October 2008 and October 2010 (Fig. 6b, Table 2).

Surveys along the Catalan and Provence coasts and in the Balearic Islands indicated that no appreciable mortality occurred after the summer period, from 2007 to 2010. In contrast, mortality records from other western Mediterranean areas by other research teams indicated that *I. fasciculata* was also injured in the southern coast of Spain (Table 1).

### Temperature conditions during the sponge die-off

The mean (± s.e.) and maximum temperatures during summer periods are summarized in Table 3. Summer mean and maximum temperatures at 15 m observed in 2008 and 2009 were among the highest values recorded in both areas during the 2007–2010 period (Table 3). Mean temperatures ranged between 22.45 and 24.4°C in Scandola RN and between 25.4 to 26.1°C in Cabrera NP; while maximum temperatures at 15 m ranged between 24.5 and 26.9°C in Scandola RN and between 26.7 and 27.7°C in Cabrera NP (Table 3).

**Table 3:** Temperature records (from 1 August to 30 September) at 15 m depth obtained in Scandola RN and Cabrera NP.

Scandola RN -15 m			
	TX	s.e.	Tmax
2007	22.45	0.1500	24.53
2008	23.55	0. 353	26.27
2009	24.40	0.100	26.90
2010	22.49	0.0437	25.30

Cabrera NP -15 m			
	TX	s.e.	Tmax
2007	25.47	0.076	26.7
2008	25.76	0.091	27.2
2009	26.17	0.139	27.7
2010	26.00	0.069	27.1

TX and T max refer to the average mean and maximum temperatures, respectively, obtained for the available period in each study area.

**Figure 7:**
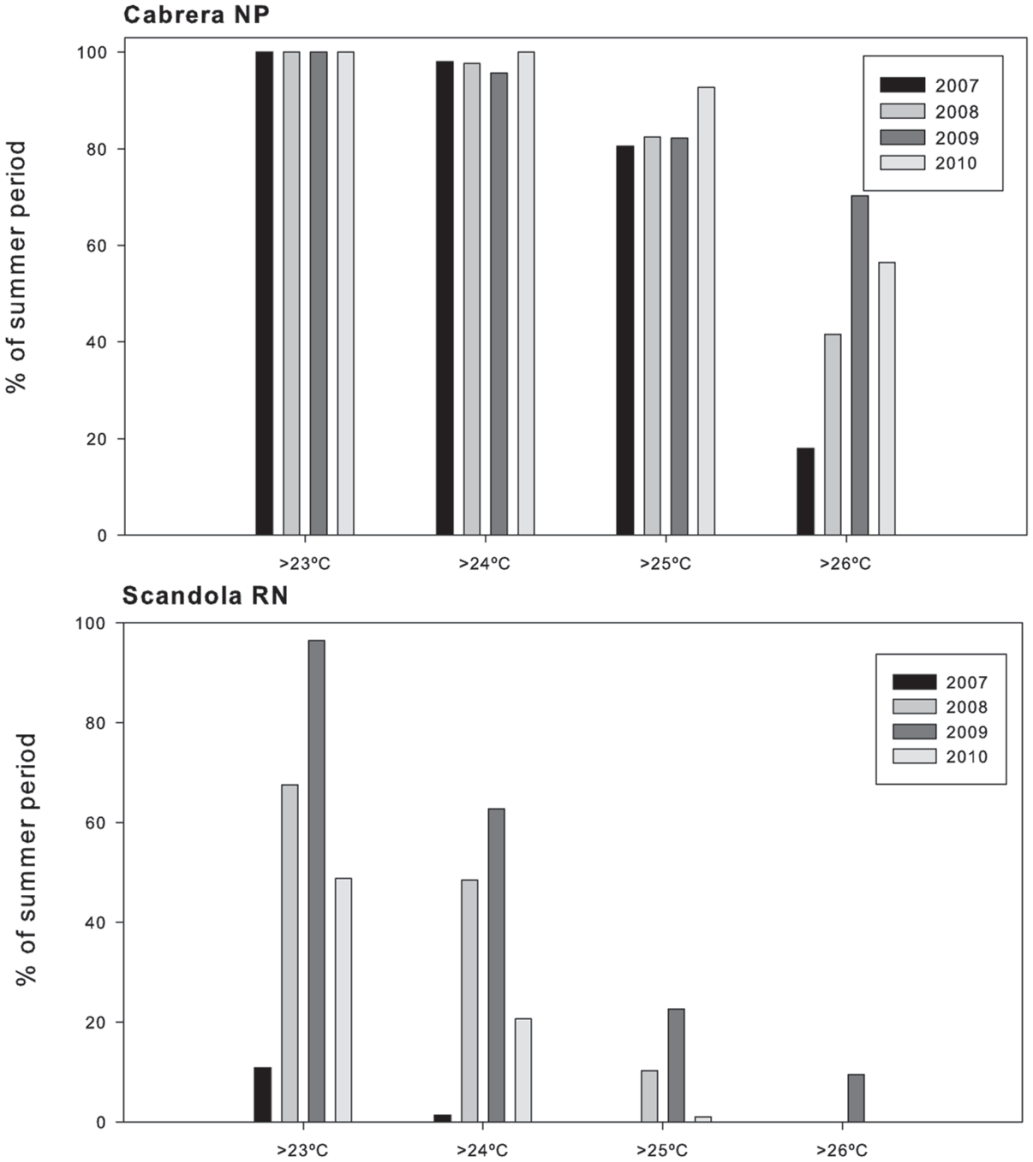
Percentage of the summer period with temperatures above 23°C, 24°C, 25°C and 26°C in Cabrera NP (a) and above 23°C, 24°C and 25°C in Scandola RN (b). Data from SOMLIT (temperature sensors) data series.

Regarding temperature thresholds, in Cabrera NP the years 2008 and 2009 displayed higher percentages of time of exposition than in 2007 regardless the temperature threshold considered, although the percentage of time above 26°C showed the largest differences. In 2010, values were similar to those of the mortality years; however by then the density of populations of *I. fasciculata* were really low (see Impact of sponge disease section) preventing the assessment of sponge mortality (Figure 7a).

In contrast, temperatures exceeded 25°C and 26°C in Scandola RN only in the mortality years (Table 3, Fig. 7b). Considering the percentages of time above 24 and 25°C, differences among years with and without sponge mortality were evident: in 2008 and 2009 the percentage of time with water temperatures above 25°C were at least two-fold than that in 2007 and 2010 (Fig. 7b). Overall, summer temperatures conditions at 15 m were much warmer, in Cabrera NP than in Scandola RN (Table 3).

### Relationship between sponge mortality and temperature

**Figure 8:**
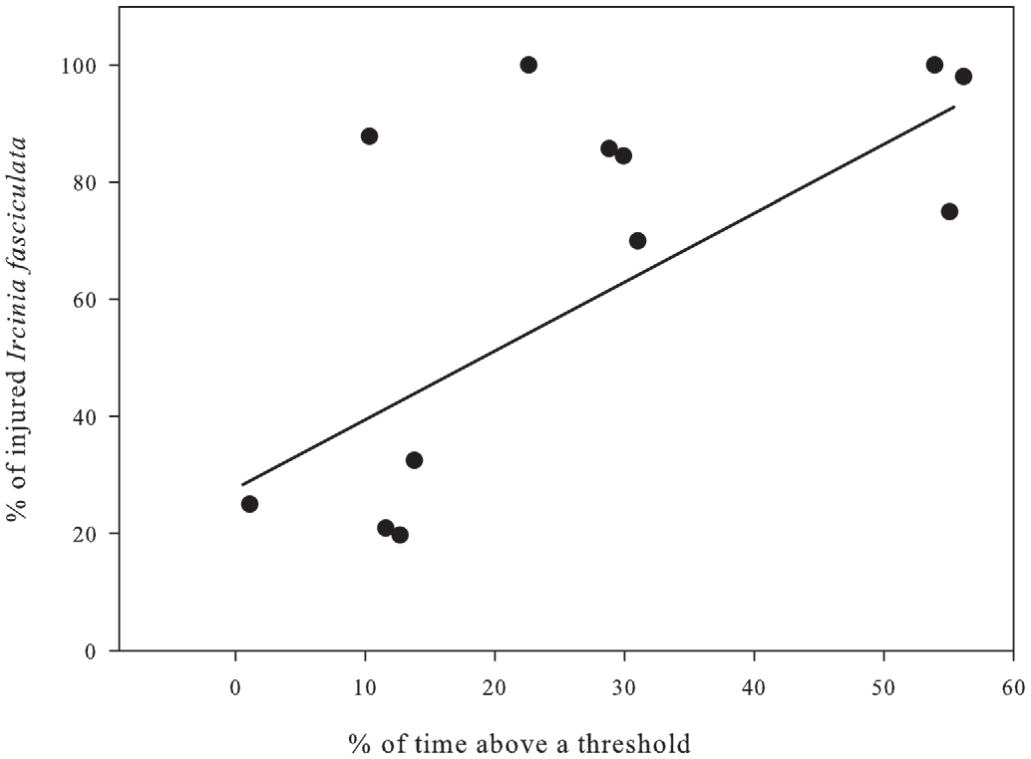
Relationship between the percentage of time above a temperature threshold (26°C) from August 1 to September 30 and the percentage of affected *I. fasciculata* colonies in Cabrera NP.

The percentage of time above 26°C for Cabrera populations and above 25°C for the Scandola population was positively correlated to the percentage of affected individuals of *I. fasciculata* for each site (Fig. 8, R = 0.672, F = 8.15, p = 0.0171). The greater percentage of summer time above a maximum threshold, the higher the impact on sponge populations (Fig. 8).

### Experimental outcome of increased temperature on the photosynthetic microsymbionts

**Figure 9:**
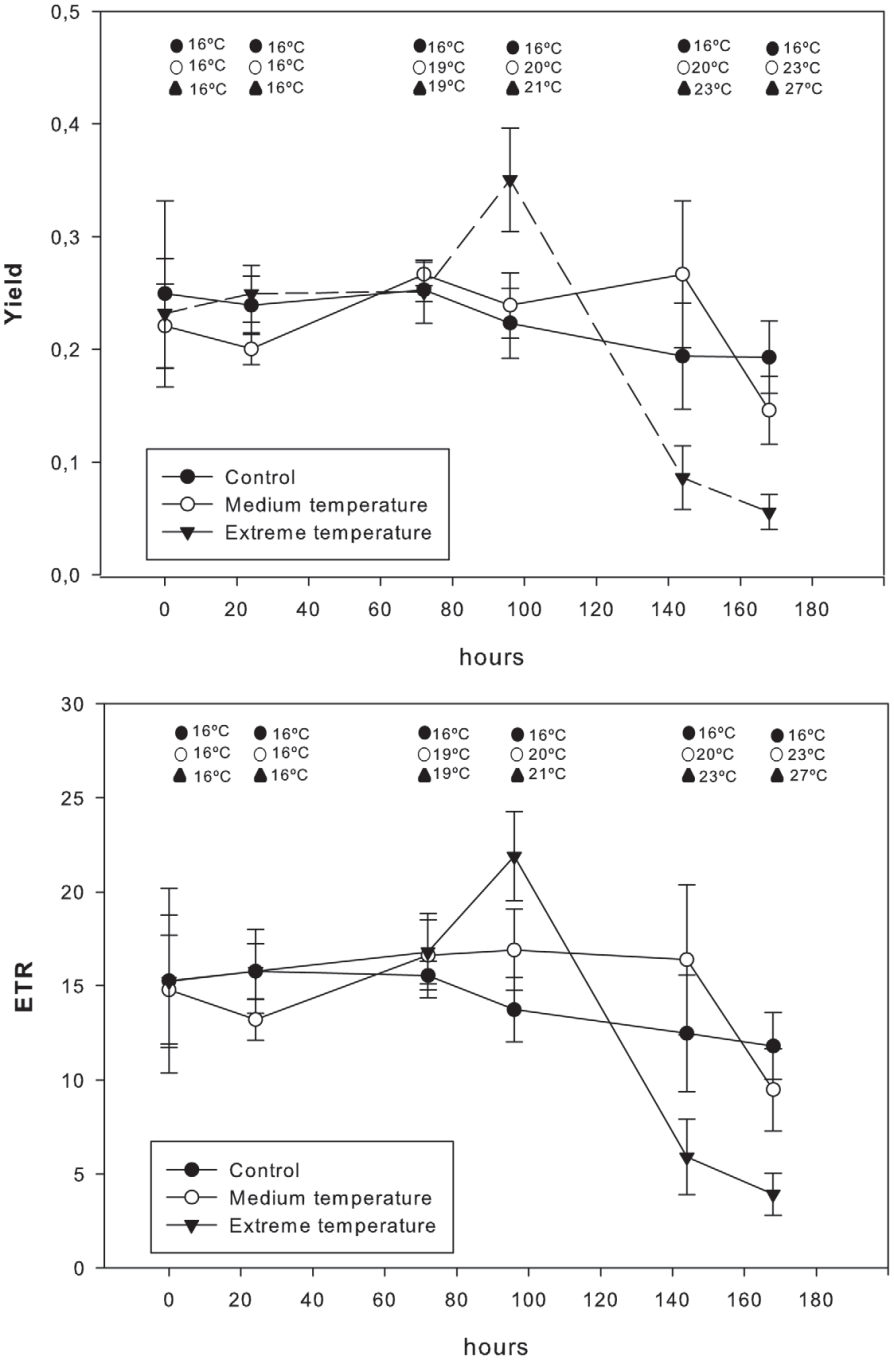
The effective quantum yield (Φ_PSII_) and photosynthetic electron transfer (ETR) during the experiment for the "control,” “medium” and “extreme” treatments. Bars represent standard errors.

The effective quantum yield (Φ_PSII_) and the photosynthetic electron transfer (ETR) were similar for all specimens at the beginning of the experiment (ANOVA F-test; p>0.1; Fig. 9). At the end of the experiment, photosynthetic parameters were significantly lower in *I. fasciculata* specimens submitted to extreme temperatures, compared to specimens submitted to low (control) and medium temperatures, which did not differ between them (Table 4, Fig. 9). Photosynthesis was inhibited in specimens submitted to extreme temperatures because both Φ_PSII_ and ETR significantly decreased to nearly zero at the end of the experiment (Table 4, Fig. 9).

**Table 4:** 1-Results of one-way ANOVA comparing initial and final fluorescence variables (Yield, ETR) for the Control, Medium and Extreme temperature treatments, and 2- results of one-way ANOVA comparing different treatments fluorescence variables (Yield, ETR) for the initial and final time point of the experiment.

		Yield			ETR		
		MS	F		MS	F	
1	Control	0.009	0.340	n.s.	32.76	0.367	n.s.
1	Medium temperature	0.018	2.591	n.s.	91.81	2.267	n.s.
1	High temperature	0.075	7.924	**	307.70	6.198	**
2	Initial time (among treatments)	0.001	0.066	n.s.	0.454	0.005	n.s.
2	Final time (among treatments)	0.021	4.566	*	81.71	4.140	*

*show significant differences p<0.05 and

**p<0.01.

## Discussion

Sponge mass mortality events occurred during the late summers of 2008 and 2009, affecting *I. fasciculata* populations thriving in shallow sublittoral habitats in central and southern parts of the western Mediterranean Sea. These mortality events affected 80 to 100% of *I. fasciculata* individuals in the studied populations. During the rest of the study period, basal mortality of *I. fasciculata* was also observed. In contrast, we did not detect significant mortality rates for *S. spinosulum* during the study period. Populations of other keratose sponge species involved in previous mortality events (e.g., *Spongia agaricina, Hippospongia communis*) [4]–[6] did not show any signs of mortality.

Our surveys detected the same mortality pattern for all populations studied, even though they were carried out in two Mediterranean areas hundreds of kilometers apart (Cabrera NP and Scandola RN). Almost all of the *I. fasciculata* individuals showed partial or total mortality. Conversely, even though *S. spinosulum* shared habitat with *I. fasciculata* in both localities, it only occasionally displayed small necrotic areas covered by a white bacterial veil.

The disease extended along the coasts of Corsica, the Balearic Islands, and the southern Spanish littoral (Granada and Chafarinas). We did not find signs of mortality in any of the other surveyed northwest Mediterranean areas (Table 1) nor in Columbretes MPA (authors obs., Diego K. Kersting, pers. comm.).

The geographical distribution of the disease strongly suggests that only the warmest areas of the western Mediterranean were affected. Mortality was observed only in areas with mean summer temperatures at least 1 to 2°C above those previously recorded on the northern Mediterranean coast [26], [31]. This result strongly contradicts the hypothesis of [32] suggesting the disease originated in the northwestern Mediterranean and only recently reached southern areas. Although significant sponge mortalities have been reported several times in the Mediterranean [5]–[6], [8], [33]–[34], this new disease episode exclusively affects *I. fasciculata*, pointing to a possible role of symbiotic cyanobacteria in the disease. Current disease features differ from those previously reported (before 2008) for other Mediterranean sponges, which start with grey or whitish zones and a bacterial veil on the sponge surface [6], [34]. In contrast, in the current mortality event, the affected sponges were characterized by small round pustules that developed into large, rounded areas of dead tissue, which led to the complete death of most specimens. The external symptoms of the current disease were more akin to those reported for *Spongia officinalis*, *Hippospongia communis communis* or *Ircinia variabilis*, which also started with the formation of white spots on the sponge surface [5], [8], [35]–[37].

Our study reports, for the first time, population descriptors (mortality incidence and densities) of sponge species before and after mortality outbreaks, allowing an accurate assessment of the impact of mortality on the species. Previous studies were carried out when mortality had already affected many individuals in the population, causing the density decline to not be properly evaluated. Shallow water populations of *I. fasciculata* were clearly decimated after the 2008 and 2009 outbreaks in both studied areas, with mortality rates similar to those reported for the most dramatic examples of sponge mortality (*Spongia* spp.) in the Caribbean Sea (70 to 95%; [38]). In contrast, in southeastern Spain (Granada coast; [32]), the impact was at least three-fold lower than in Cabrera NP and Scandola RN (present study). Bearing in mind that affected sponges fall apart and disappear about one month after displaying the first signs of injury ([23]–[24]; authors obs.), this inter-regional difference could be attributed to mortality underestimation in the Granada area, where surveys were carried out two months after the 2008 outbreak [32].

Recovery of the impacted populations is uncertain. It has been reported that when a small proportion of the sponges are affected, populations are able to recover [8]. In contrast, when mortality rates are high (similar to the values reported here) or if other mortality sources are acting synergistically (e.g., overfishing), sponge populations may disappear [39]. As a result, we may predict a dramatic extinction of the populations studied. However, only continued monitoring of the affected and unaffected populations will allow us to elucidate the long-term consequences of these events, which may not only harm sponge populations but also alter benthic assemblages.

Previous massive sponge mortality events in the Mediterranean have been related to high temperatures [4]–[6], [33]. Our temperature records also indicate that the *I. fasciculata* outbreak coincided with particularly high seawater temperatures recorded in 2008 and 2009 in Cabrera NP and in 2008 in Scandola RN (Table 3 and Fig. 5). We found a significant positive relationship between the mortality rate and the percentage of time that sponge populations experienced temperatures above a certain threshold (Fig. 7). In contrast, Maldonado et al. (2010) studied the same mortality outbreak in *I. fasciculata* populations but did not find any relationship between temperature and mortality incidence. However, in the later study, the data used to search for a temperature-disease relationship were sea surface temperature (SST) from satellite retrievals, which likely are not representative of the temperatures experienced by sponge populations *in situ* [26]. Interestingly, northern unaffected areas (Marseilles, Port Cros, Medes Islands, Blanes; www.t-mednet.org and data available from the authors) also reached warm-temperature conditions at 15 m depth in 2008 and in 2009, but temperatures never surpassed 24°C, which commonly occurred in the affected zones. This observation points to 24°C as the threshold for triggering *I. fasciculata* disease. However, *I. fasciculata* populations from Cabrera NP were often subjected to temperatures above 24°C in other years (Figure 7a), and only when temperatures exceed the 26°C threshold are clear-cut inter-annual differences observed. Meanwhile, in Scandola RN, temperatures above 26°C were rarely reached (Figure 7b and [26]), suggesting a differential temperature threshold for triggering mortality in the Cabrera NP and Scandola RN populations. It has been already demonstrated that different populations of the same species subjected to contrasting temperature regimes can show differential resilience to global warming [40]. The acquisition of a long-term series of biological and temperature data will allow us to better establish the linkage of mortality events with future temperature conditions.

The other surveyed species, *S. spinosulum*, was not significantly affected in 2008 and 2009 or other more recent mass mortality events [4]. Individuals of this species have larger sizes and higher abundances in the warmer waters of the central and eastern Mediterranean than in the colder western Mediterranean [20], while individuals of *I. fasciculata* are of similar size in colder and warmer Mediterranean regions. This positive relationship between maximum sponge size and water temperature suggests that *S. spinosulum* is better adapted to withstand higher temperatures than *I. fasciculata.*

Although current and previous outbreaks of *I. fasciculata* are positively related to high temperatures, the primary cause of the disease remains controversial. Exposure to high temperatures may cause partial or total death of specimens as a result of physiological stress [41]–[44], a decrease in the efficacy of defense mechanisms [45], [46], and/or the development of pathogens [17], [47]–[48]. However, sponges more frequently affected by periodic diseases belong to the so-called “bacteriosponge” category because they harbor abundant *a priori* symbiotic bacteria; future research should focus on changes in the composition of the symbiotic bacterial community under different temperature conditions. In fact, [49] reported shifts in the composition of the microsymbiotic community in tropical sponges subjected to elevated temperatures, and they attributed sponge mortality to the loss of symbionts and the consequent establishment of alien microbial populations, including potential pathogens.

*I. fasciculata* and *S. spinosulum* populations showed significantly different responses to the mass mortality events analyzed in this study. To explain this differential impact, the working hypothesis was that high temperatures might induce a breakdown of the cyanobacteria-sponge symbiosis present in *I. fasciculata*, but not in *S. spinosulum*. The decay of cyanobacteria might play a key role in sponge mortality because these symbionts seem to greatly contribute to the carbon requirements of the sponge [50]–[54].

However, the sponge-cyanobacteria symbiosis also implies biochemical reactions with harmful consequences for the sponge. Elevated temperatures may lead to an increase in reactive oxygen species (ROS) [55] and to a lower antioxidant efficiency [56]. This ROS increase has been related to the presence of tissue necrosis (white patches on the ectosome) in the Mediterranean sponge, *Petrosia ficiformis,* after a period of calm, warm water [57], which may be a plausible hypothesis to explain the present *I. fasciculata* disease.

Our experiment demonstrated that high temperatures result in a significant reduction in photosynthetic efficiency (Fv/Fm) and ETR of the symbiotic cyanobacteria, with consequent inhibition of photosynthesis (Fig. 9). The TEM observations revealed degeneration or even total absence of cyanobacteria on the affected sponge tissues, while heterotrophic bacteria were present in both affected and unaffected tissues. A particular bacterial type that was only present in the affected tissues is ultrastructurally similar to that reported for tunneling spongin plates and dead skeletal fibers of both healthy sponges and exposed spongin fibers. Thus, it seems to be an opportunistic microorganism that consumes skeletal material of dead sponges, as suggested by [8] and [58], rather than the primary cause of infection, as proposed in [32]. Although a dysfunction in cyanobacterial functioning cannot be considered the only cause of sponge mortality, impairment of sponge fitness due to increasing temperatures might trigger the virulence of pathogens that would otherwise have remained inactive or few in numbers. Furthermore, symbiotic relationships have evolved under constant environmental conditions, and thus, slight changes in environmental variables may prompt their imbalance. In this sense, invertebrates that harbor symbionts, such as corals and sponges, seem to be particularly threatened by warming [59]–[61], which causes these organisms to become more susceptible to pathogenic agents when exposed to a warming climate [2], owing to basic modifications in their biological properties.

In the present study, we were able to quantify devastating mortalities in the sponge *I. fasciculata*, leading to quasi-local extinctions of the studied populations. The current mortality event is the most severe reported to date in the Mediterranean.

Because future climatic projections for the Mediterranean region clearly indicate warming, with increases in extreme temperatures and heat waves [62]–[64], new mass mortality events are extremely likely to occur during the next several decades. Sponges have been demonstrated to be very sensitive to global change perturbations (e.g. [34], [49], [65]–[67]). In particular, the so-called bacteriosponges are especially vulnerable to environmental changes that may cause imbalances in their delicate symbiotic relationships with microbes. These massive sponges play a structural role in benthic assemblages, and their alteration may threaten rich Mediterranean biodiversity.
